# Toxic Effects of Methylene Blue on the Growth, Reproduction and Physiology of *Daphnia magna*

**DOI:** 10.3390/toxics11070594

**Published:** 2023-07-07

**Authors:** Shuhui Li, Yixin Cui, Min Wen, Gaohua Ji

**Affiliations:** 1 National Demonstration Center for Experimental Fisheries Science Education, Shanghai Ocean University, Shanghai 201306, China; 1911202@st.shou.edu.cn; 2 Engineering Research Center of Environmental DNA and Ecological Water Health Assessment, Shanghai Ocean University, Shanghai 201306, China; m210100130@st.shou.edu.cn (Y.C.); m200100099@st.shou.edu.cn (M.W.)

**Keywords:** antimicrobial dyes, zooplankton, life table parameters, oxidative damage

## Abstract

Methylene blue (MB) is a disinfectant used in aquaculture to prevent and treat fish diseases. However, the release of MB can pose a risk to the receiving water bodies. Zooplankton are the most sensitive organisms among aquatic life. Hence, this study examined the acute and chronic toxic effects of MB on zooplankton using *Daphnia magna* (*D. magna*) as a test organism to provide basic data for risk assessment. The results show that 48 h-EC_50_ and 24 h-LC_50_ were 61.5 ± 2.3 and 149.0 ± 2.2 μg/L, respectively. Chronic exposure to MB affected the heart rate, beat frequency of the thoracic limbs, and reproductive ability of *D. magna* at environmental concentrations higher than 4.7 μg/L. The cumulative molts, time to production of the first brood, and total number of living offspring were affected at different MB concentrations, while “abortions” were observed in high-exposure groups. The activity of superoxide dismutase was increased, while glutathione S-transferase activity was stimulated at low concentrations and inhibited at high concentrations. In addition, the malondialdehyde content increased with increasing concentrations of MB. Our findings demonstrate the impact of MB on the reproduction and growth of freshwater species, as well as their physiological responses. These results have implications for establishing guidelines on the use of MB in aquaculture and setting discharge standards.

## 1. Introduction

Methylene blue (MB) is an aromatic dye with the molecular formula C_16_H_18_CIN_3_S and a benzene ring with redox characteristics. It is commonly used in various industries, including the textile, pharmaceutical, paper, dyeing, printing, paint, pharmaceutical, and food industries, as well as in medical research and aquaculture [1,2,3].

In aquaculture, MB is a popular environmental disinfectant. Disinfectants frequently used in aquaculture can be broadly categorized into three groups: halogens, dyes, and surfactants. Among the dyes, trityl methane dyes such as malachite green and crystal violet are prohibited substances due to their long residual time and toxic side effects, so MB has emerged as one of the best alternatives. It is commonly used to disinfect aquaculture environments because the ionic compound generated in the aqueous solution can compete with microbial enzyme systems for hydrogen ions and inactivate the enzymes, which results in the loss of viability of microorganisms. Furthermore, MB, as an antifungal drug, is used to prevent and control fish diseases such as saprolegniasis, ichthyophthiriasis, chilodonelliosis, and gill disease to reduce mortality in fish during transportation [4]. 

The environment of aquaculture water directly affects whether aquatic animals can grow quickly and healthily, and the quality of the water is closely related to the occurrence of diseases. As the water quality deteriorates, it encourages the production of different types of pathogens, endangering the growth and development of farmed animals. Disinfectants play a crucial role in aquaculture, from cleansing water bodies to bathing fish before transferring them to ponds, disease management, and even water quality regulation. Disinfectants should be used rationally and scientifically to eradicate or destroy pathogenic microorganisms in the aquaculture environment and stop the spread of disease.

Malachite green is listed as a banned drug in some countries [5] due to its potential toxicity to the water environment and human health. It belongs to the same group of tritylene-based dyes as methylene blue and has a long residual time, as well as teratogenic, carcinogenic, and mutagenic risks [6]. The widespread use of methylene blue can result in residues in water bodies that persist in the environment [7], and the associated water contamination problems cannot be ignored. Additionally, the color of malachite green can prevent sunlight from passing through the water body, resulting in reduced dissolved oxygen and inhibited photosynthesis [8]. This can lead to reduced diversity in biological communities [9,10,11,12] and interfere with the normal functioning of aquatic ecosystems.

Recent studies have reported varying toxic effects of MB on different aquatic organisms (Supplementary Materials, Table S1). Perlberg et al. [13] found that MB was teratogenic to *Pterophyllum scalare*, reporting that exposure to a 5 ppm concentration resulted in a higher incidence of non-inflatable swim bladders. In contrast, Soltanian et al. [14] found that goldfish (*Carassius auratus*) exposed to a 2 mg/L solution of methylene blue for 21 days had significantly reduced lethality caused by *Aeromonas hydrophila*. However, the fish also exhibited significantly lower levels of neutrophils and aspartate aminotransferase, indicating some immunosuppressive effects and potential harm to their health. Comparing the toxicity of MB in various aquatic animals, it is evident that although it is a valuable tool in aquaculture, it still has some negative consequences. Currently, the U.S. Food and Drug Administration (FDA), EU Directive 96/23/EC, and Japan’s “positive list” have established guidelines for detecting MB residues in aquatic products [15]. Despite being repeatedly banned, MB continues to be used due to its low cost and high efficiency [16,17]. In addition, many countries, such as China, have yet to establish limits on the use of methylene blue in animal-derived food products.

Freshwater zooplankton are a critical component of aquatic environments, playing a vital role in the material cycle and energy flow. Despite their importance, there are still unanswered questions regarding the ecotoxicity of MB in this group of organisms. *Daphnia magna* is a typical representative of zooplankton [18], with a transparent body. It feeds on algae, which helps improve water quality, and is a natural bait for filter-feeding fish [19,20]. *D. magna* lays eggs in its brood chamber, where they develop until the eye point appears, forming an embryo. The embryo then develops into a neonate and is released from the parent’s body (Supplementary Materials, Figure S1).

*Daphnia magna* has been extensively used for toxicity testing, identifying water pollution, and creating water quality standards due to its sensitivity to chemical exposure [21,22]. To assess the toxicity of chemicals to *D. magna*, a range of parameters were determined, including reproduction, physiology, swimming behavior, and biochemistry. Growth and reproductive capacity are sensitive indicators of chronic toxicity for *D. magna* [23] and are also important factors in assessing population growth capacity [24], including parameters such as lifespan, total number of living offspring, time to first brood, cumulative molts, reproductive rate, and number of aborted eggs. Swimming behavior parameters consist of swimming activity, swimming time, swimming speed, and so on. Among the physiological parameters are feeding rate, heart rate, thoracic limb activity, post-abdominal claw movement, and compound eye activity. Biochemical parameters can be categorized as enzymatic or non-enzymatic. Quantitative studies on enzyme activity variations in *D. magna* have been conducted to determine the ecological risk of chemicals [25,26,27].

The safety of the aquatic environment is a pressing issue in the 21st century, with significant ramifications for society and the global economy. Yee et al. [28] highlighted the persistent issue of the negative effects of man-made surface biological contamination. To address this issue, we used *D. magna* as a test organism to investigate the acute and chronic toxic effects of MB and calculated the median effect concentration (EC_50_), median lethal concentration (LC_50_), and no observed effective concentration (NOEC) to provide basic data for aquatic ecological risk assessment. Additionally, our findings provide a reference for the safe and rational use of MB and ecological diversity conservation.

## 2. Materials and Methods

### 2.1. Chemicals

The MB solution used in this study was purchased from Sangon Biotech Company (Shanghai, China), with a blue color and purity ≥ 98%. The basic physical and chemical properties of MB are presented in Table 1. A stock solution with a concentration of 100 mg/L was prepared with ultrapure water and stored at 4 °C in a refrigerator protected from light.

### 2.2. Preparation of Daphnia magna

*D. magna* were cultured in the laboratory for over 3 generations, all derived from a single parent. They were cultured in a 500 mL glass beaker placed in an incubator at 20 ± 1 °C, with a 16:8 light/dark photoperiod and a light intensity of 3000 Lux. EPA medium was used as the culture medium, and it was renewed 2–3 times a week. The animals were fed daily on *Chlorella vulgaris* (at a concentration of 2.5 × 10^6^ cells/mL), and dead *D. magna* and impurities at the bottom were cleaned daily. Larger, more active females with more eggs were selected 24 h before the experiment and incubated in a separate beaker. The newly produced juveniles (6~24 h) were used as test organisms.

### 2.3. Acute Experiments

In 48 h immobilization toxicity tests, referring to the Test No. 221 guidance document [29], 6 concentration groups (30, 39.6, 52.3, 69, 91.1, and 120 μg/L) and a control group were set up according to the pilot experiment. The test system consisted of 50 mL beakers containing 30 mL of MB solution. Ten healthy neonates (<24 h) were randomly placed in each beaker without feeding, and three replicates were set up for each group. After 48 h, the morphology of *D. magna* was observed under a microscope, and the inhibition rate was calculated (inhibited being defined as having a heartbeat but not swimming). The 48 h-EC_50_ value was obtained through curve fitting.

In 24 h lethality toxicity tests, after pilot experiments, 6 treatment groups (110, 124, 140, 157, 177, and 200 μg/L) and a control group were established. The test system consisted of 50 mL beakers containing 30 mL of MB solution. Ten healthy neonates (<24 h) were randomly placed in each beaker without feeding, and three replicates were set up for each group. After 24 h, the morphology of *D. magna* was observed under a microscope, and the mortality rate was calculated (with the criterion of no heartbeat as death). The 24 h-LC_50_ value was obtained by curve fitting.

### 2.4. Chronic Experiments

According to the value of 24 h-LC_50_, 6 treatment groups (1.5, 2.7, 4.7, 8.4, 15, and 26.7 μg/L) and one blank control group were set up using the equal logarithmic spacing method. The experimental system consisted of 50 mL beakers containing 30 mL of MB solution, with one healthy neonate (<24 h) randomly placed in each beaker, and 10 replicates were set up for each concentration group, for a total of 70 neonates (7 × 10) used in the experiment, which lasted 21 days. *Chlorella vulgaris* were fed daily at a density of 2.5 × 10^6^ cells/mL. During the experiment, a semi-static exposure test was used, and the exposure solution was replaced every 2 days. The growth and reproductive status were observed and recorded daily, including cumulative molts, time of the first brood, number of offspring in the first brood, total number of broods, and number of living offspring per brood, to calculate the intrinsic growth rate (r_m_) of the population. The newborns were promptly removed after birth.

At the end of the experiment, each *D. magna* was placed in the groove of a single concave glass slide, and an appropriate amount of MB exposure solution was dropped in. The body length (from the helmet to the front end of the tail spine, excluding the tail spine), the heart rate, and the frequency of thoracic limb movement were measured using a stereomicroscope and VistarImage software. Each *D. magna* was measured 3 times.

### 2.5. Measurement of Physiological Parameters

Antioxidant damage tests were conducted on *D. magna* in the 8.4, 15, and 26.7 μg/L exposure concentrations and the blank control group based on the results of the chronic test. For these tests, 100 healthy neonates (age 6~24 h) were randomly placed in a 250 mL beaker containing 200 mL of MB solution, without feeding, and there were 3 replicates for each group. After 24 h, the *D. magna* were collected, washed 3 times, and transferred to a 1.5 mL tube containing 0.81 mL of physiological saline. The tube was then placed at −40 °C for 12 h and then at 4 °C for 1 h to thaw. An ultrasonic cell disrupter was used to crush the tissue. The prepared 10% tissue homogenate was centrifuged at 2500 rpm for 15 min in a high-speed frozen centrifuge at 4 °C. The supernatant obtained was the crude enzyme solution for measurement. Superoxide dismutase (SOD) activity, glutathione S-transferase (GST) activity, and malondialdehyde (MDA) content were measured using test kits purchased from Nanjing Jiancheng Bioengineering Institute, Nanjing, China.

### 2.6. Statistical Analysis

The experimental data were analyzed and statistically processed using SPSS 23.0 and Excel software. The experimental results were subjected to linear or nonlinear fitting analysis using OriginPro 9.1 software. The most suitably fitted model was selected based on the R^2^ value of the fitting curve being closer to 1 and the *p* value being smaller. The trend of inhibition and mortality of *D. magna* under exposure to different concentrations of methylene blue was fitted using the Hill function (Equation (1)) to obtain the 48 h-*EC_50_* and 24 h-LC_50_ values:

(1) $$f\left(x\right)=\frac{1}{1+{\left(\frac{EC50}{x}\right)}^{m}}$$

where *f*(*x*) refers to the mortality (or immobilization rate) of *D. magna*, *x* refers to the concentration of MB solution (μg/L), and m refers to the curve shape parameter.

The Log3P1 model (Equation (2)) was used to fit the trend of heart rate and thoracic limb activity in the chronic treatment:

(2) y=a−bln(x+c)

where *y* refers to the heart rate or beat frequency of the thoracic limb (times/min), *x* refers to the concentration of MB solution (μg/L), and *a*, *b* and *c* are constants.

The intrinsic rate of population increase (*r_m_*) was initially calculated by Equation (3), and then the precise value was obtained through the stepwise approximation method in Equation (4) [30,31]:

(3) $$r_{m}=\frac{\ln R_{0}}{T},\;\;R_{0}=\sum_{0}^{\infty }m_{x}l_{x},\;\;T=\frac{\sum_{0}^{\infty }xl_{x}m_{x}}{R_{0}}$$

(4) $$\sum_{x=0}^{n}e^{-rm_{x}}l_{x}m_{x}=1$$

where *x* is the age of *D. magna* (d), *lx* is the survival rate at age *x*, *m_x_* is the fecundity at age *x*, *R*_0_ is the net reproduction rate, and *T* is the generation time (d). 

The normality of the parameters was tested using the Shapiro–Wilks test. A one-way ANOVA followed by an LSD post hoc test was used to analyze the differences between the blank control and various MB concentration groups, and the experimental results were presented as mean ± standard error.

## 3. Results

### 3.1. Acute Immobilization Toxicity Tests

**Figure 1:**
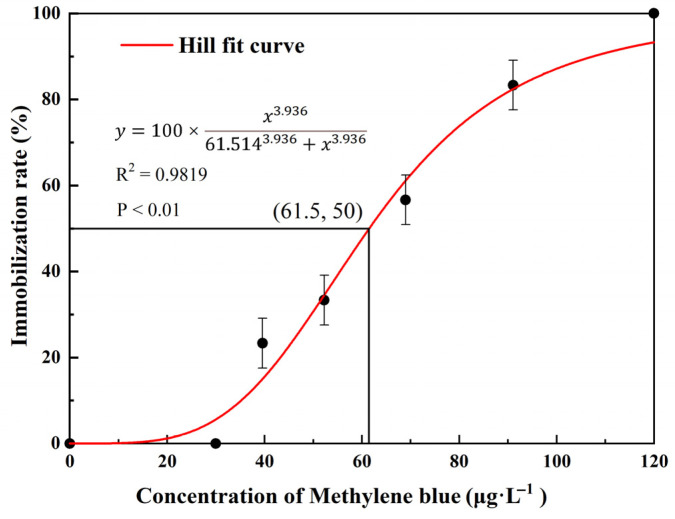
Trend of immobilization rate fitted by Hill function in acute toxicity tests. Means and standard errors are shown.

The immobilization rate of *D. magna* increased with increasing MB concentration (Figure 1). The 48 h-EC_50_ of MB for *D. magna* was determined to be 61.5 ± 2.3 μg/L.

### 3.2. Acute Lethality Toxicity Tests

**Figure 2:**
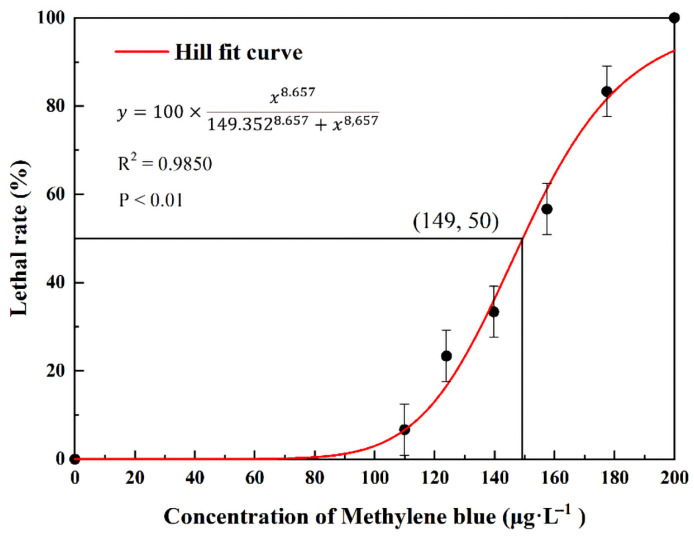
Trend of mortality rate fitted by Hill function in acute toxicity tests. Means and standard errors are shown.

The mortality of *D. magna* increased with the increased MB concentration (Figure 2). The 24 h-LC_50_ of MB for *D. magna* was determined to be 149.0 ± 2.2 μg/L.

### 3.3. Damage Caused by MB to D. magna Bodies in Acute Toxicity Tests

**Figure 3:**
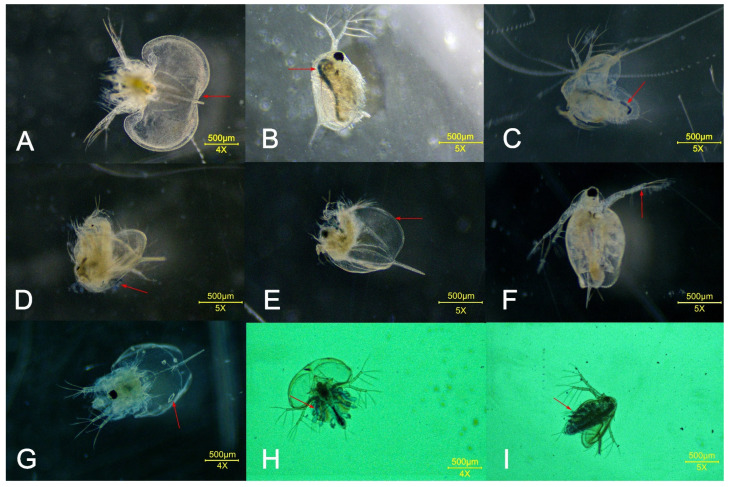
Damage to *D. magna* caused by MB in acute toxicity tests: (**A**) swollen and whitish carapace; (**B**) blue intestines; (**C**) swollen carapace and blue intestines; (**D**) carapace stuck with blue substance; (**E**) swollen carapace and partial loss of thoracic limb; (**F**) blue substance sticking to second antenna; (**G**) body turned blue throughout, with swollen carapace and holes; (**H**) swollen carapace and blue substance sticking to thoracic limb; (**I**) blue deposits in intestines and thoracic limbs.

In the acute exposure test, the tested *D. magna* suffered from various degrees of damage. Some individuals had holes in their carapace and lost their thoracic limbs, while others had a swollen carapace and blue residue on their antennae, appendages, intestines, and carapace. The bodies of *D. magna* that had stopped beating were white and sank to the bottom of the exposure solution (Figure 3).

### 3.4. Chronic Toxicity of Methylene Blue in D. magna

#### 3.4.1. Heart Rate and Thoracic Limb Activity

**Figure 4:**
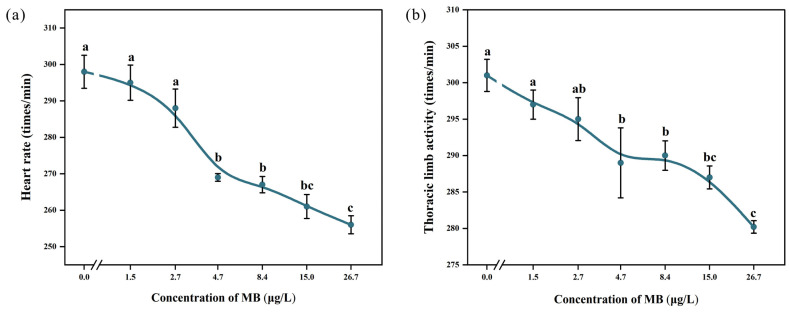
Effect of MB on (**a**) heart rate and (**b**) thoracic limb activity of *D. magna* (mean ± SE, *n* = 10); significant differences (*p* < 0.05) between different treatment groups and the control group are indicated by different letters.

The heart rate of *D. magna* decreased as the MB concentration increased (Figure 4). At 4.7 μg/L, the heart rate of the exposed group (269 ± 2.35 times/min) was significantly different (*p* < 0.05) from that of the control group (298 ± 10.15 times/min). The lowest heart rate was observed in the 26.7 μg/L MB treated group (256 ± 5.57 times/min), which was significantly different (*p* < 0.05) from the control group, as well as the 1.5, 2.7, 4.7, and 8.4 μg/L MB treated groups. Similarly, as the MB concentration increased, the thoracic limb beat frequency decreased. At 4.7 μg/L, the beat frequency of the exposed group (289 ± 10.75 times/min) was significantly different (*p* < 0.05) from that of the control group (301 ± 4.95 times/min). The lowest beat frequency was observed in the 26.7 μg/L exposed group (280.2 ± 1.92 times/min), which was significantly different (*p* < 0.05) from that of the control group as well as the 1.5, 2.7, 4.7, and 8.4 μg/L treatment groups.

**Figure 5:**
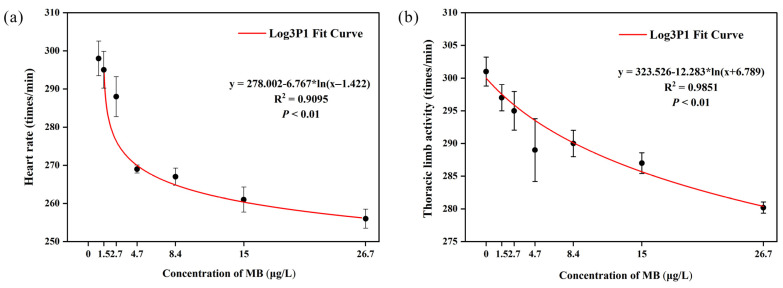
Effects of MB on (**a**) heart rate and (**b**) thoracic limb activity of *D. magna* after fitting with the Log3P1 curve. Means and standard errors are shown.

The inhibitory effect of MB on heart rate and thoracic limb beat frequency was dose-dependent (Figure 5).

#### 3.4.2. Body Length

**Figure 6:**
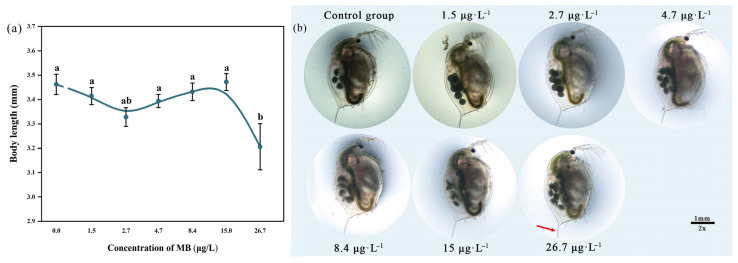
Effect of MB on (**a**) body length and (**b**) morphology of *D. magna* in each group at 21 days (mean ± SE, *n* = 10); significant differences (*p* < 0.05) between different treatment groups and the control group are indicated by different letters.

After exposure to methylene blue for 21 days, the body length of *D. magna* in the 15 μg/L group increased (3.472 ± 0.078 mm) compared to the control group (3.462 ± 0.094 mm), but there was no significant difference (*p* > 0.05). The body length of *D. magna* in all other groups was shorter than that of the control group, and the body length in the highest concentration group (26.7 μg/L) was significantly shorter (3.206 ± 0.211 mm, *p* < 0.05; Figure 6).

#### 3.4.3. Reproduction

**Figure 7:**
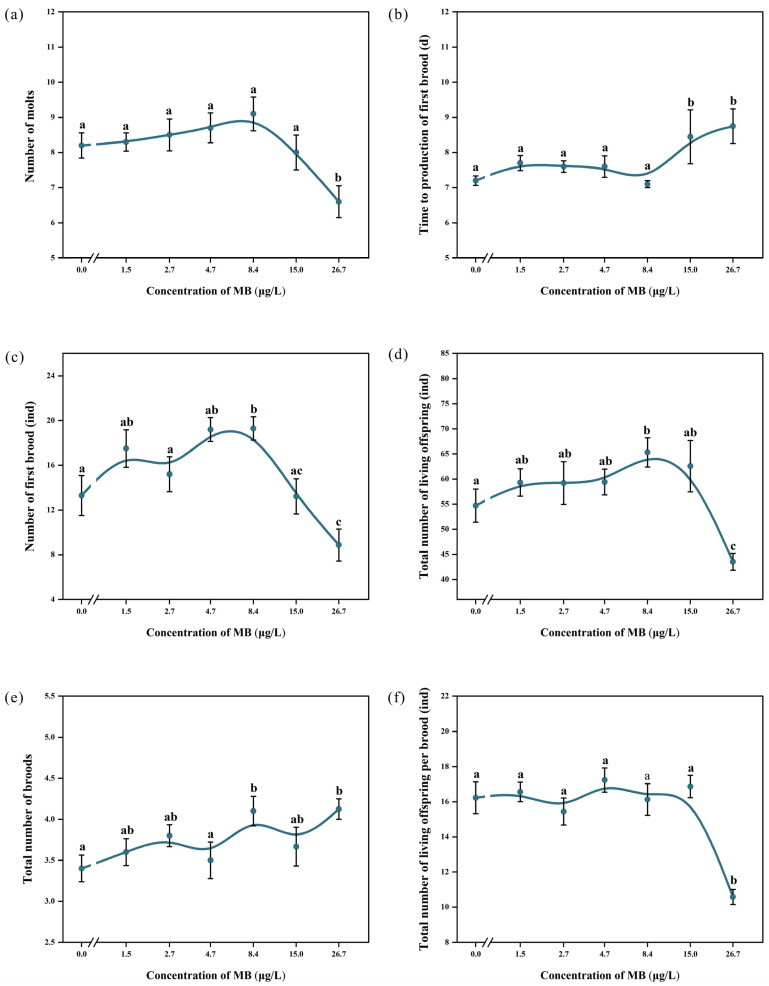
Influences of MB on (**a**) number of molts, (**b**) time to production of first brood, (**c**) number of first brood, (**d**) total number of living offspring, (**e**) total number of broods, and (**f**) total number of living offspring per brood (mean ± SE; *n* = 10). Significant differences (*p* < 0.05) among groups are indicated by different letters.

The total number of molts of *D. magna* showed a trend of increasing and then decreasing with increasing MB concentration. When the MB concentration was 8.4 μg/L, the number of molts reached a peak at 9.1 ± 1.52 times, but there was no significant difference (*p* > 0.05) compared with the control group (8.2 ± 1.14 times) (Figure 7a). When the concentration was higher than 8.4 μg/L, the number of molts began to decrease. The total number of molts in the group exposed to the highest concentration of 26.7 μg/L (6.6 ± 1.43 times) was significantly less than that of the control group (*p* < 0.05).

Except for the 8.4 μg/L exposure group, which had a slightly earlier first brood time than the control group (7.1 ± 0.316 days versus 7.2 ± 0.422 days), the first brood time in all other groups was delayed compared to the control group. In particular, the first brood time of *D. magna* in the 15 and 26.7 μg/L exposure groups (8.4 ± 2.297 and 8.75 ± 1.389 days, respectively) was significantly later than that of the control group (*p* < 0.05) (Figure 7b).

**Figure 8:**
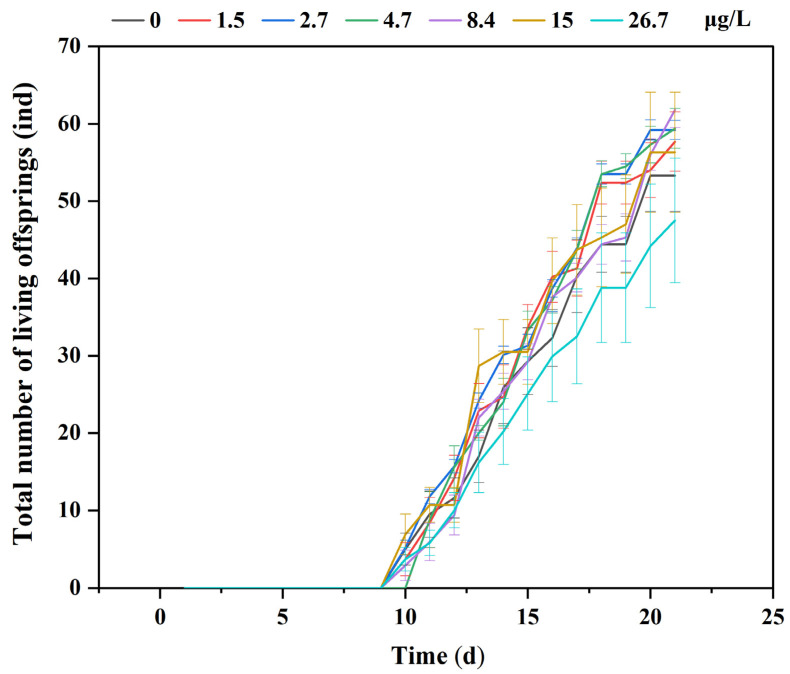
Cumulative number of living offspring over a 21-day period. Means and standard errors are shown.

The number of first brood neonates and total offspring produced by *D. magna* showed an initial increase followed by a decrease with increasing MB concentration. The highest number of first brood neonates (19.3 ± 3.302) and total offspring produced (65.3 ± 9.21) were observed in the 8.4 μg/L concentration group, which was significantly higher than the control group (*p* < 0.05). In contrast, the lowest number of first brood neonates (8.875 ± 4.051) and total offspring produced (43.5 ± 4.75) was observed in the highest concentration group (26.7 μg/L), which was significantly lower than the control group and other exposure groups (*p* < 0.05; Figure 7c,d). The number of broods produced in all exposure groups was higher compared to the control group, and the number was significantly higher in the 8.4 and 26.7 μg/L groups than the control group (*p* < 0.05), with 4.1 ± 0.57 and 4.1 ± 0.35 broods, respectively (Figure 7e). The average number of neonates produced per brood decreased with increasing MB concentration, and all exposure groups had fewer neonates per brood than the control group. The lowest average number of neonates produced per brood was observed in the highest concentration group (26.7 μg/L), with 10.58 ± 1.23, which was significantly lower compared to the control group (*p* < 0.05; Figure 7f). The cumulative number of living offspring in each group over 21 days is shown in Figure 8.

**Figure 9:**
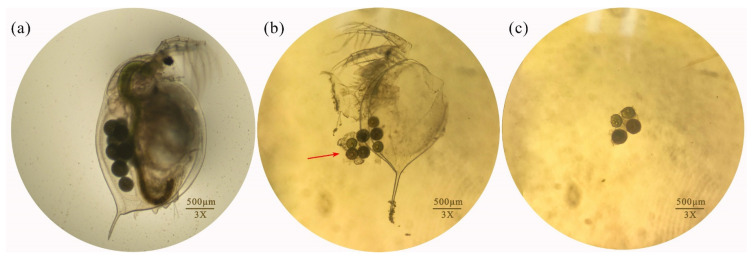
(**a**) Control group of *D. magna* and aborted eggs in groups exposed to (**b**) 15 and (**c**) 26.7 μg/L observed under a stereomicroscope (3×).

In addition, “abortion” was observed during the experiment (Figure 9). One *D. magna* in the 15 μg/L exposure group aborted seven eggs on the seventh day. The undeveloped eggs fell off the brood chamber and adhered to the molted carapace. Another individual in the 26.7 μg/L exposure group aborted four eggs on the fifth day, and the eggs sank to the bottom of the beaker.

**Figure 10:**
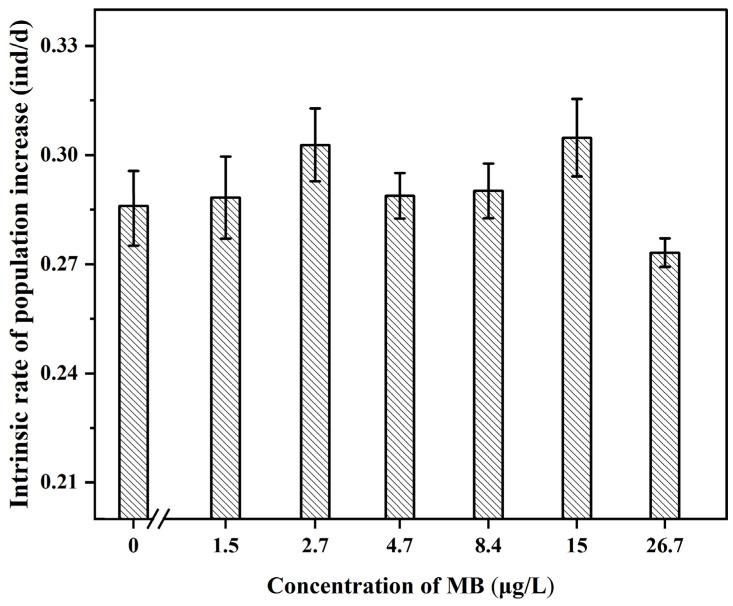
Intrinsic rate of population increase of *D. magna* (mean ± SE; *n* = 10).

As the concentration of MB increased, the intrinsic growth rate showed a trend of first increasing and then decreasing (Figure 10). The intrinsic growth rate was higher in the groups exposed to 1.5, 2.7, 4.7, 8.4, and 15 μg/L of methylparaben than the control group, while the rate was lower in the 26.7 μg/L group than the control group, but the difference was not significant (*p* > 0.05).

### 3.5. Antioxidant Enzymes, Detoxification Enzymes, and Oxidative Damage Markers

**Figure 11:**
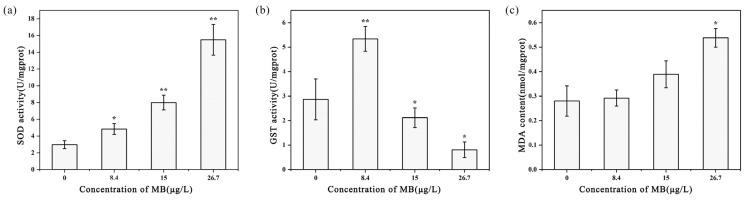
Effects of MB on (**a**) SOD activity, (**b**) GST activity, and (**c**) MDA content of *D. magna* (mean ± SE; *n* = 3; * *p* < 0.05 between the different treatment groups and the control group; ** *p* < 0.01 between the different treatment groups and the control group).

The changes in superoxide dismutase (SOD) activity in *D. magna* tissues after exposure to MB are shown in Figure 11a. The SOD activity of the control group was the lowest, at 2.973 ± 0.479 U/mgprot. With increasing MB concentration, the SOD activity gradually increased. The SOD activity in *D. magna* exposed to 8.4 μg/L MB was significantly higher than that in the control group (*p* < 0.05). The SOD activity in *D. magna* exposed to 15 μg/L MB was 7.996 ± 0.878 U/mgprot, significantly higher than that in the control group (*p* < 0.01). When the MB concentration was 26.7 μg/L, the SOD activity of *D. magna* was the highest (15.497 ± 1.83 U/mgprot), which was 5.21 times that of the control group, and there was a significant difference (*p* < 0.01).

As the concentration of MB increased, GST activity in *D. magna* tissues was first induced and then inhibited. When the MB concentration was 8.4 μg/L, the GST activity was the highest (5.338 ± 0.505 U/mgprot), which was 1.86 times that in the control group (2.868 ± 0.835 U/mgprot), and the induced GST activity was significant (*p* < 0.01). However, when the MB concentration exceeded 8.4 μg/L, the GST activity significantly decreased compared to the control group (*p* < 0.05). This was evidenced by the GST activity in the *D. magna* tissues exposed to 15 and 26.7 μg/L MB, which was 2.119 ± 0.403 and 0.808 ± 0.319 c U/mgprot, respectively (Figure 11b).

As the concentration of MB increased, the MDA content in *D. magna* tissues also increased. However, there was no significant difference in MDA content between the 8.4 and 15 μg/L exposure groups and the blank control group (*p* > 0.05), with values of 0.292 ± 0.033 and 0.389 ± 0.055 nmol/mgprot, respectively. In contrast, the MDA content in *D. magna* exposed to the highest concentration of 26.7 μg/L (0.538 ± 0.038 nmol/mgprot) was 1.92 times higher than that in the control group (0.28 ± 0.062 nmol/mgprot) (Figure 11c).

## 4. Discussion

EC_50_ and LC_50_ are commonly used to assess the toxicity of pollutants to aquatic organisms. Previous studies reported 24 h-LC_50_ values of MB of 5.769 mg/L for *Litopenaeus vannamei* [32] and 31.60 mg/L for *Limnodrilus* [33], suggesting that the sensitivity to MB is highest in *D. magna* and that MB is a potent toxin for zooplankton. Furthermore, the EC_50_ and LC_50_ values vary depending on the type of pollutant. Abe et al. [30] reported EC_50_ values of two azo dyes, basic red 51 (BR51), a synthetic dye, and erythromycin (Ery), a natural dye, on *D. magna* of 0.10 mg/L (0.09–0.11) and 19.7 mg/L (15.7–24.9), respectively. Similarly, Verma [34] found that the 48 h-EC_50_ values of the azo dyes Remazol Parrot Green and Remazol Golden Yellow for *D. magna* were 55.32 and 46.84 mg/L, respectively, while Kanhere [35] reported that the 48 h-EC_50_ of malachite green was 0.77 mg/L. Compared with these results, *D. magna* was found to be more sensitive to the toxicity of methylene blue, a thiazide dye. Based on the United Nations Globally Harmonized System of Classification and Labelling of Chemicals (GHS Rev. 9, 2021) for acute aquatic toxicity (class I: 48 h-EC_50_ ≤ 1 mg/L; class II: 1 mg/L < 48 h-EC_50_ ≤ 10 mg/L; class III: 10 mg/L < 48 h-EC_50_), the toxicity of MB to *D. magna* falls into class I.

The heart rate and beat frequency of the thoracic limbs of *D. magna* are important indicators of their overall health, reflecting the status of their feeding, respiration, metabolism, and endocrine system [36]. Several chemicals have been found to affect *D. magna*’s heart rate and beat frequency [37,38]. In this experiment, higher MB concentrations were found to decrease the heart rate and beat frequency of the thoracic limbs in *D. magna*. This may be due to the accumulation of MB with prolonged exposure, resulting in high toxicity and difficulty degrading it [39]. Long-term accumulation of MB in *D. magna* may lead to physiological abnormalities, decreased heart rate, and diminished eating capacity. Similar findings were reported by Eghan et al. [40], who observed time- and dose-dependent suppression of heart rate and thoracic limb beat frequency in *D. magna* exposed to acrylamide. Bownik et al. [41] also reported a time- and dose-dependent decrease in heart rate and thoracic limb beat frequency in response to ketoprofen, while procaine penicillin caused a concentration-dependent depression of heart rate and beat frequency [42]. *D. magna* has a myogenic heart, which means the myocardial contractions are not influenced by brain activity [43,44,45,46]. Pirtle et al. [45] suggested that the autonomic beating of *D. magna’s* heart relies on hyperpolarization activating T-type calcium channels and cyclic nucleotide-gated ion channels. MB alters the activity of these ion channels, leading to changes in heart rate, which could explain the observed decrease in heart rate in *D. magna*. A decreased heart rate implies reduced O_2_ and nutrient supply to cells [47], which impacts lymphatic blood circulation and immune response [48], thereby affecting the beat frequency of the thoracic limbs. 

The thoracic limbs, which are the feeding organs of *D. magna*, are covered with bristles that filter food from the water and deliver it to the mouth. A decrease in the beat frequency of the thoracic limbs can affect the water filtration rate of *D. magna*, reducing their feeding capacity. Food intake is crucial for energy replenishment, and feeding capacity is closely related to individual growth and reproduction ability [49]. Moreover, decreased beat frequency of the thoracic limbs may be associated with the intestinal contents. On the one hand, it can be caused by an increase in particulate matter, including food [50,51]. Lari et al. [50] studied the effect of oil sands process-affected water (OPSW), a by-product of bitumen extraction, on *D. magna* and found that the exposed group exhibited a change in the color of the intestine from green to brown, a significantly higher density of algal cells compared to the control group, and a simultaneous decrease in the beat frequency of the thoracic limbs. On the other hand, exposure to dissolved toxicants can lead to decreased Na^+^/K^+^-ATPase activity in *D. magna*, resulting in reduced transmission between thoracic limb neurons and muscles and, in turn, decreased beat frequency of the thoracic limbs [36]. Additionally, blue residue was observed in the intestines of exposed *D. magna*, partially obstructing the flow of intestinal contents and potentially contributing to reduced feeding capacity.

Chronic exposure to MB significantly impacted the growth and reproductive capacity of *D. magna*. Specifically, the 26.7 μg/L exposure group exhibited significantly shorter body length and fewer molts than the control group, indicating that MB had some developmentally toxic effects, strongly inhibiting the growth and development of *D. magna*, as it normally develops through molting.

Regarding reproduction, exposure to high concentrations of MB caused a significant delay in the production of the first brood. At medium MB concentrations, the number of the first brood and total number of living offspring significantly increased, while at higher concentrations they decreased, indicating hormesis, a stimulative effect at low concentrations, and an inhibitory effect at high concentrations [52]. The total number of broods was higher in all exposure groups than in the control group, but the number of living offspring per brood was lower in the exposure groups. When considering the 21-day reproduction results, the increase in the number of the first brood and total number of living offspring in the 8.4 μg/L group was due to the earlier sexual maturation time, while the decrease in the 26.7 μg/L group was due to a significant delay in sexual maturation and a decrease in the number of living offspring per brood. This adaptive response to the environment aligns with previous studies, suggesting that *D. magna* deliberately increase the number of reproductions and reduce the number of single reproductions to stabilize the population in adverse environments [53].

Many studies have demonstrated the occurrence of hormesis in the growth, reproduction, and swimming behavior of *D. magna* when exposed to various contaminants. For instance, low concentrations of bioplastics promoted the reproductive rate, while higher concentrations inhibited it [54]. Similarly, low concentrations of ciprofloxacin and ofloxacin were found to stimulate a shortening of the first oogenesis time and an increase in brood size in *D. magna* [55]. In studies investigating the effects of fluoxetine and propranolol on *D. magna* swimming activity [56], intermediate drug doses (1~10 μg/L) significantly promoted swimming activity, while high doses (>100 μg/L) had the opposite effect, causing a significant decrease in swimming activity.

The decreased reproductive capacity of *D. magna* in the high-concentration groups may be attributed to the allocation of energy, primarily used for growth, reproduction, and basal metabolism. The stress response triggered by MB elevated the basal metabolic energy consumption of *D. magna*, thereby reducing the available energy reserves for growth and reproduction [57]. Consequently, there was a decline in reproductive ability and, in certain instances, even the occurrence of “abortion”. These findings are consistent with those of a previous study [57].

The intrinsic rate of population increase is a measure of the population’s ability to expand under ideal conditions. In this study, the intrinsic rate of population increase initially showed an increasing trend with increasing MB concentration, followed by a decreasing trend. However, these trends were not significantly different from those observed in the control group. The concentration of energy allocated to basal metabolism in *D. magna* under the stress of MB may influence its growth and reproduction, contributing to the observed pattern. The sensitivity of reproduction parameters to MB varied, and the order of sensitivity for these indicators was as follows: number of first brood = total number of broods = total number of living offspring > time to production of first brood > cumulative molts = number of living offspring per brood.

SOD plays a vital role in *D. magna*’s antioxidant defense system by eliminating reactive oxygen species (ROS) through the catalysis of superoxide anion radicals (O_2_^−^) into hydrogen peroxide (H_2_O_2_) and oxygen (O_2_) [58]. This enzyme exerts a protective effect on the cells of the organism. Assessing SOD activity in *D. magna* can provide insights into its ability to adapt to MB exposure. In our study, SOD activity exhibited a continuous increase with escalating MB concentration. SOD activity in the highest concentration group was 5.21 times higher than that in the control group. These findings indicate that under the stress of MB, *D. magna* consistently enhanced its antioxidant capacity in response to the increasing assault of ROS and the unfavorable environment.

Excess reactive oxygen radicals in *D. magna* tissues that cannot be scavenged by SOD can lead to lipid peroxidation of cell membranes, causing cellular damage. This damage can be assessed by measuring the MDA content. In our study, the MDA content in *D. magna* exhibited a positive correlation with increasing MB concentration and was significantly higher compared to the control group, indicating oxidative damage. Despite the increased SOD activity, the MDA content also increased, suggesting that SOD was unable to fully eliminate all ROS. It is noteworthy that the highest MB concentration tested did not inhibit SOD activity, as reported in previous studies by Shen et al. [59] and Duan et al. [60] using different compounds but demonstrating similar effects. The elevated MDA levels in our study imply that MB exposure stimulated the generation of intracellular ROS in *D. magna*, leading to oxidative alterations of cellular components.

GST is an essential detoxifying enzyme that plays a crucial role in scavenging free radicals and facilitating detoxification processes. It catalyzes the conjugation of harmful endogenous or exogenous substances with reduced glutathione (GSH), forming more soluble and nontoxic derivatives that can be efficiently excreted or broken down by enzymes [61]. GST also possesses the ability to scavenge excess ROS, limit lipid peroxidation, and mitigate oxidative stress-induced damage [62]. In this study, GST activity exhibited hormetic effects, with induction observed in the low-concentration group and inhibition in the high-concentration group. At low concentrations of MB, GST activity increased to 1.86 times that of the control group, effectively scavenging free radicals and serving a detoxification function. However, at high MB concentrations, GST activity declined rapidly, possibly due to the depletion of intracellular GSH content. Consequently, toxins accumulated, disrupting the balance between free radical production and elimination and leading to the inactivation of GST, impairing its normal participation in the detoxification reaction. This finding is consistent with previous research [59], which demonstrated that after 24 h of dibutyl phthalate exposure, GST activity in *D. magna* neonates was dramatically elevated at 0.5 mg/L and reduced at 2 mg/L.

The influence of MB on *D. magna* involves complex physiological and biochemical processes, and our study examined only its effects on growth and reproduction. Consequently, there remains a knowledge gap concerning the toxicological mechanism of MB. Future investigations could bridge this gap by integrating conventional toxicological analysis with ecotoxicological genomics data, including transcriptomics, proteomics, metabolomics, and epigenomics. This comprehensive approach would enable a thorough exploration of the effects of MB on *D. magna* and other zooplankton species [63].

## 5. Conclusions

Methylene blue exhibited pronounced toxicity toward *D. magna*, with increasing toxic effects correlating with increasing concentration. Chronic exposure to MB significantly affected the growth and reproduction of *D. magna*, with heart rate and thoracic limb beat frequency proving to be more sensitive indicators than body length. Furthermore, MB could induce antioxidant stress in *D. magna*. The maximal concentration of MB at which no adverse effects were observed (NOEC) was determined to be 4.7 μg/L. Establishing water quality criteria for MB primarily relies on zooplankton, particularly *D. magna*, which is the most sensitive species to MB contamination (Supplementary Materials, Figure S2).

## Figures and Tables

**Table 1:** Physical and chemical properties of methylene blue (MB).

Chemical Name	Molecular Formula	Molecular Weight	CAS Registry Number	Solubility (g/L)	Chemical Structure
Chloro-3,7-bis(dimethylamino) phenothiazine-5-buzz-trishydrate	C_16_H_18_CIN_3_S·3 (H_2_O)	373.9	7220-79-3	50 (20 °C)	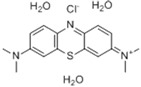

## Data Availability

The data presented in this study are available upon request from the corresponding author.

## Supplementary Materials

The following supporting information can be downloaded at: https://www.mdpi.com/article/10.3390/toxics11070594/s1, Table S1. Acute toxicity of methylene blue to aquatic animals. Figure S1. Developmental stages of Daphnia magna under the stereomicroscope. Figure S2. Logistic fitting curve of species mean acute values (SMAV) of methylene blue. References [32,33,64,65,66,67,68] are cited in the supplementary materials.
