# Supplementary material for: Toxic Effects of Methylene Blue on the Growth, Reproduction and Physiology of Daphnia magna
Source: Toxics. 2023 Jul 7;11(7):594. doi: 10.3390/toxics11070594 (PMC10384865; doi:10.3390/toxics11070594)
Supplement: Supplementary file 1 [file toxics-11-00594-s001.zip › toxics-2404293-supplementary.pdf]

toxics

*Supplementary Materials*

# Toxic Effects of Methylene Blue on the Growth, Reproduction and Physiology of *Daphnia magna*

**Shuhui Li [1,†], Yixin Cui [2,†], Min Wen [2] and Gaohua Ji [1,2,*]**

**Table S1.** Acute toxicity of methylene blue to aquatic animals.

| Experimental Organism | Test Results | Reference |
| --- | --- | --- |
| *Megalobrama terminalis* | 24h $LC_{50}$： 20.22 mg/L<br>48h $LC_{50}$： 11.90 mg/L<br>96h $LC_{50}$： 8.89 mg/L | (Dai Y et al. 2020) |
| *Litopenaeus Vannamei* | 24h $LC_{50}$: 5.769 mg/L<br>48h $LC_{50}$: 4.698 mg/L | (Zhou G et al. 2020) |
| *Rhodeus ocellatus* | 24h $LC_{50}$: 45.30 mg/L<br>48h $LC_{50}$: 18.76 mg/L | (Chen W et al. 2010) |
| *Limnodilus hoffineisteri* | 24h $LC_{50}$: 31.60 mg/L<br>48h $LC_{50}$: 20.60 mg/L<br>96h $LC_{50}$: 18.80 mg/L | (Yin H et al. 2007) |
| *Pimephales promelas* | 96h $LC_{50}$: 45.00 mg/L(20℃）<br>96h $LC_{50}$:: 15.00 mg/L(25℃) | (Rifici et al. 1996) |
| *Penaeus californiensis* | 1h $LC_{50}$: 100 mg/L | (Hanks 1976) |
| *Salmo trutta* | 24h $LC_{50}$: 54 mg/L<br>48h $LC_{50}$: 32.8 mg/L | (Willford WA et al. 1966) |
| *Salvelinus fontinalis* | 24h $LC_{50}$: 49.8 mg/L<br>48h $LC_{50}$: 22.9 mg/L | |
| *Salvelinus namaycush* | 24h $LC_{50}$: 35 mg/L<br>48h $LC_{50}$: 34 mg/L | |

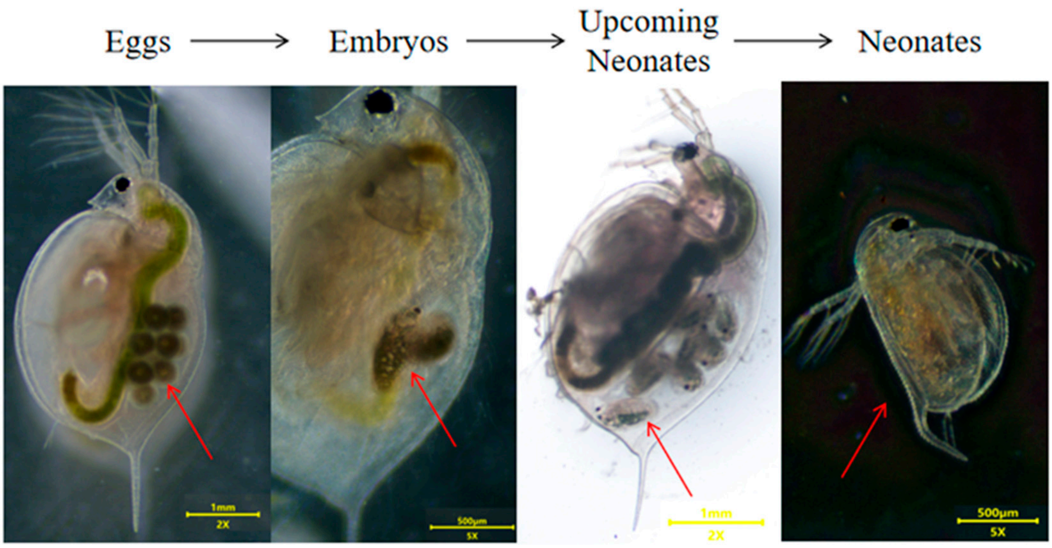


**Figure S1.** Developmental stages of *Daphnia magna* under the stereomicroscope.

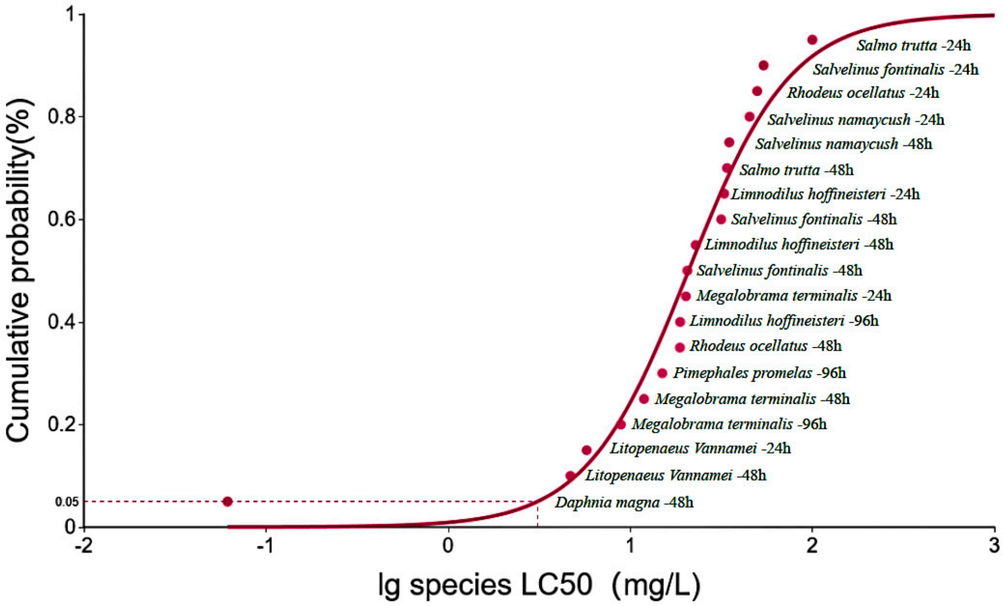


**Figure S2.** Logistic fitting curve of species mean acute values (SMAV) of methylene blue.

## References


1. Dai Y., Xie N., Ma H.; et al. Acute toxicity test of copper iron mixture, Metrifonate, methylene blue and Povidone-iodine on summerlings of *Megalobrama terminalis* [J]. Fisheries Science & Technology Information, 2020, 47(4):4.DOI:10.16446/j.fsti.20190900124.
2. Zhou G., Ma R., Yang Z.; et al. Acute Toxicity of Prometryn, Phoxim, and Methylene Blue and Their Histopathological Effects on *Penaeus vannamei* [J]. Asian Journal of Ecotoxicology, 2020, 15(06): 279-89.
3. Chen, W., Qu, J., Zhou, F. Study on the acute toxicity of methylene blue to young *Rhodeus Ocellatus* [J]. Guangdong Agricultural Sciences, 2010, 37(05): 131-2.
4. Yin H, Kan X, Shi X; et al. The acute poisoning function of methyl to *Limnodilus hoffineisteri* [J]. Natural Science Journal of Harbin Normal University, 2007, (4): 80-2.
5. Rifici L M, Cherry D S, Farris, J. L.; et al. Acute and subchronic toxicity of methylene blue to larval fathead minnows (*Pimephales promelas*): Implications for aquatic toxicity testing [J]. Environmental Toxicology and Chemistry, 1996, 15(8): 1304-8.

6. Hanks K S. Toxicity of some chemical therapeutics to the commercial shrimp, *Penaeus californiensis* [J]. Aquaculture, 1976, 7: 293-4.
7. Willford WA;USDOI, FWS, Bur Sport Fish Wildl; Invest Fish Control No.18, Resourc Publ No.35: 10 (1966) as cited in the ECOTOX database. Available from, as of November28, 2022. [J.].
